# Characterization of Two Marine Lignin-Degrading Consortia and the Potential Microbial Lignin Degradation Network in Nearshore Regions

**DOI:** 10.1128/spectrum.04424-22

**Published:** 2023-04-12

**Authors:** Yvette Ley, Xiao-Yu Cheng, Zhi-Yue Ying, Ning-Yi Zhou, Ying Xu

**Affiliations:** a State Key Laboratory of Microbial Metabolism, Joint International Research Laboratory of Metabolic and Developmental Sciences, and School of Life Sciences and Biotechnology, Shanghai Jiao Tong University, Shanghai, China; Swansea University

**Keywords:** lignin biodegradation, lignin-derived aromatics, ligninolytic enzymes, marine consortia, nearshore regions

## Abstract

Terrestrial organic carbon such as lignin is an important component of the global marine carbon. However, the structural complexity and recalcitrant nature of lignin are deemed challenging for biodegradation. It has been speculated that bacteria play important roles in lignin degradation in the marine system. However, the extent of the involvement of marine microorganisms in lignin degradation and their contribution to the oceanic carbon cycle remains elusive. In this study, two bacterial consortia capable of degrading alkali lignin (a model compound of lignin), designated LIG-B and LIG-S, were enriched from the nearshore sediments of the East and South China Seas. Consortia LIG-B and LIG-S mainly comprised of the *Proteobacteria* phylum with *Nitratireductor* sp. (71.6%) and *Halomonas* sp. (91.6%), respectively. Lignin degradation was found more favorable in consortium LIG-B (max 57%) than in LIG-S (max 18%). Ligninolytic enzymes laccase (Lac), manganese peroxidase (MnP), and lignin peroxidase (LiP) capable of decomposing lignin into smaller fragments were all active in both consortia. The newly emerged low-molecular-weight aromatics, organic acids, and other lignin-derived compounds in biotreated alkali lignin also evidently showed the depolymerization of lignin by both consortia. The lignin degradation pathways reconstructed from consortium LIG-S were found to be more comprehensive compared to consortium LIG-B. It was further revealed that catabolic genes, involved in the degradation of lignin and its derivatives through multiple pathways via protocatechuate and catechol, are present not only in lignin-degrading consortia LIG-B and LIG-S but also in 783 publicly available metagenomic-assembled genomes from nine nearshore regions.

**IMPORTANCE** Numerous terrigenous lignin-containing plant materials are constantly discharged from rivers and estuaries into the marine system. However, only low levels of terrigenous organic carbon, especially lignin, are detected in the global marine system due to the abundance of active heterotrophic microorganisms driving the carbon cycle. Simultaneously, the lack of knowledge on lignin biodegradation has hindered our understanding of the oceanic carbon cycle. Moreover, bacteria have been speculated to play important roles in the marine lignin biodegradation. Here, we enriched two bacterial consortia from nearshore sediments capable of utilizing alkali lignin for cell growth while degrading it into smaller molecules and reconstructed the lignin degradation network. In particular, this study highlights that marine microorganisms in nearshore regions mostly undergo similar pathways using protocatechuate and catechol as ring-cleavage substrates to drive lignin degradation as part of the oceanic carbon cycle, regardless of whether they are in sediments or water column.

## INTRODUCTION

Marine sediment is one of the largest reservoirs of microbial diversity and organic carbon on the planet, while the microbial-driven organic carbon conversion is a major component of the global carbon cycle (1, 2). The volume of organic carbon in the nearshore region is relatively high compared to other oceanic regions. Although nearshore oceanic regions make up only 7 to 10% of the total ocean area, 80% of the total organic carbon preserved in marine sediments is found in these regions (3). Furthermore, nearshore regions also host the most diverse interaction between microbes and organic matters, facilitating active material transformation (1, 3). Structural polymers such as lignin, cellulose, and hemicellulose account for approximately one-third of the sedimented oceanic organic matter (4, 5). In particular, lignin, which is a polymer composed of three primary monolignol units: coniferyl alcohol (G), sinapyl alcohol (S), and *p*-coumaryl alcohol (H) linked together via various C-C and C-O-C bonds (6–8), is the second most abundant biomass in nature (9). However, due to its high molecular weight, relative insolubility, and complex aromatic structure, lignin is highly recalcitrant to be degraded (10, 11).

To date, several terrestrial microbes in fungal species such as *Basidiomycete* fungi, have been reported to be able to degrade lignin into simpler molecules (12–14). Most fungal lignin degraders were able to secrete extracellular ligninolytic enzymes such as laccase (Lac), class II peroxidase (lignin peroxidase [LiP], manganese peroxidase [MnP], and versatile peroxidase [VP]), or dye decolorizing peroxidase (DyP) (15), which catalyzed the hydroxylation, decarboxylation, or demethylation of lignin to produce low-molecular-weight aromatics (16). Among them, Lac and MnP directly oxidize phenolic lignin components, whereas LiP and VP oxidize the recalcitrant nonphenolic lignin components (15). DyP, on the other hand, is a kind of peroxidase found prominently in bacteria that is involved in bacterial lignin degradation (17). Although DyP is also found in plants, animals, and fungi, its roles in the microbial degradation of lignin were mostly reported in bacteria compared to fungi. In particular, there are four classifications of DyP (type A, B, C, and D), three of which are primarily from a bacterial origin (17). It has been suggested that DyP could be regarded as the bacterial equivalent of a fungal lignin-degrading enzyme. Subsequently, the produced lignin-derived single-ring aromatics can be degraded via known central degradation pathways such as catechol or protocatechuate degradation pathways by many functionally identified terrestrial bacteria (18–20). As to archaea, only the uncultured Bathyarchaeota from the marine sediments was reported to be capable of utilizing lignin for its enrichment, but the metabolic mechanism is yet to be known (21).

Presently, the involvement of bacteria in initial lignin degradation remains poorly understood. Only a few bacteria from Actinobacteria and α- and γ-Proteobacteria phyla (22, 23) were isolated from the terrestrial regions, such as Sphingomonas paucimobilis SYK-6 (20, 24), Rhodococcus jostii RHA1 (25, 26), and Pseudomonas putida (25). They have been reported for their ability to degrade lignin, but with no identification of the degradation mechanism. Limited reports from the marine environment also suggested similar findings. For instance, the Proteobacteria phylum was enriched when microbial consortia extracted from the Eastern Mediterranean Sea when treated with lignin (27), and another study showed the dominance of *Proteobacteria* during the isolation of bacteria strains from a sunken wood sample in the marine environment below the sea level of 495 m (23). However, the presence of fungi and archaea were very low in relative abundance compared to bacteria in the lignin-adapted consortia (27) and fungal degraders exhibited lower diversity and less activity in the marine (23) and aquatic system (28). As an organic carbon source, the amount of lignin storage in the marine system especially at nearshore regions was relatively lower than the amount of organic carbon input (4). Thus, it has been speculated that bacteria probably play important roles in the microbial biodegradation of lignin there, in addition to the potential degraders from fungi or archaea.

In summary, the understanding of the lignin biodegradation mechanism is mainly derived from the terrestrial environment where fungi are the vital component (15). The involvement of marine microorganisms especially bacteria in the degradation of lignin together with its derived compounds and their contribution to the oceanic carbon cycle remains elusive (29). To further understand the microbial involved in marine lignin conversion, we aimed to functionally screen bacterial consortia from the marine sediments and identify the key metabolic pathway that marine microorganisms undergo. The search for bacteria lignin degraders in the marine system was expanded, resulting in two lignin-degrading consortia LIG-B and LIG-S obtained from continental margin sediments of the East and South China Seas, respectively. The functional characterization of lignin degradation by each consortium was analyzed, including their role in the degradation stages of the various lignin-derived compounds on both the genomic and physiological levels. Moreover, the abundance of key catabolic genes in global similar regions was analyzed. Thus, this study will broaden our understanding of how microorganisms drive the oceanic carbon cycle via lignin decomposition.

## RESULTS AND DISCUSSION

### Enrichment and diversity of two lignin degradation consortia.

After five cycles of enrichment in artificial seawater medium (ASW) with alkali lignin (0.4%) as the sole carbon source, two bacterial consortia designated LIG-B and LIG-S were successfully enriched from East China and South China Seas, respectively. The relative abundances of these two consortia based on their metagenomes (SRR19975620 and SRR19975619) were analyzed. The top species at phylum and genus levels were displayed for optimized visualization, and the remaining species were merged into the Unclassified and/or Others (Fig. S1 in the supplemental material). Only bacteria were found in the microbial composition of both consortia LIG-B and LIG-S. At the phylum level, Proteobacteria accounted for the highest proportion at 91.94% and 96.36% of consortia LIG-B and LIG-S, respectively, and the second highest abundance in both consortia was sorted under unclassified (7.78% and 2.37%). Furthermore, the other classified phyla each took up no more than 0.8% in consortium LIG-B and no more than 1.08% in LIG-S.

At the genus level, the compositions and abundances of the two consortia vastly differ. The highest abundance of the dominant genus in consortia LIG-B and LIG-S was *Nitratireductor* sp. (71.59%) and *Halomonas* sp. (91.55%), respectively. The next highest abundance in both consortia was sorted under Unclassified and Others (8.86% and 10.38% in LIG-B, and 2.69% and 2.24% in LIG-S). *Mesorhizobium* (2.46%), *Martelella* (1.16%), *Rhizoboium* (1.05%), and *Chelativorans* (1.01%) were the species with a relatively higher proportion in LIG-B, and all the other classified genera each took up less than 1% in both consortia. This suggests that strains from the above highly abundant species may play key roles in lignin degradation by each consortium. However, we cannot exclude the possibility that strains from other genera with lower abundance in the consortia might also be involved in degrading lignin or lignin-derived aromatic compounds.

Similar results were also obtained in several lignin-adapted microbial consortia isolated from Eastern Mediterranean Sea (27) and sunken wood sample (23), in which bacteria were suggested to be responsible for lignin degradation in the marine system and fungi and archaea were very low in relative abundance (27). These reported marine environments are probably more suitable for the growth of lignin degraders from bacteria rather than from fungi or archaea. Moreover, there are chances of horizontal gene transfer between different species as well as the uptake of extracellular DNA material in the oceanic system, contributing to the increased ability of lignin degradation of bacteria in the marine system (30, 31).

### Lignin degradation and decolorization by consortia.

**FIG 1:**
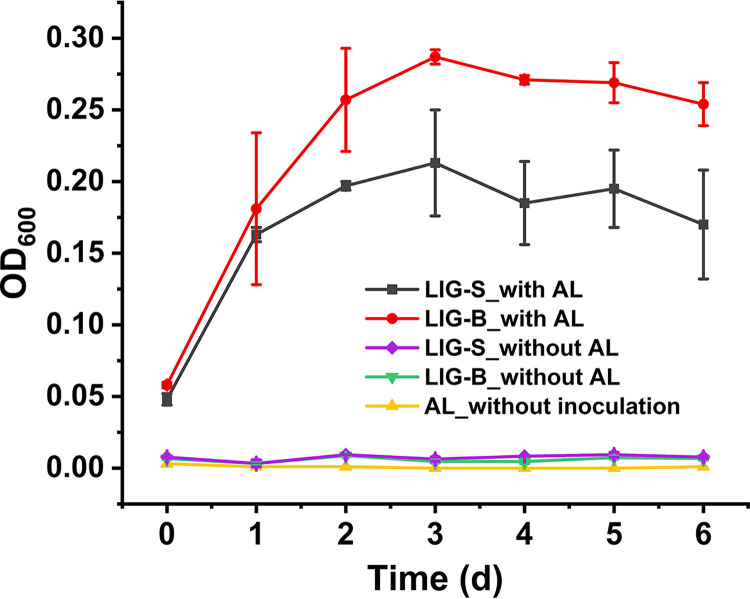
Growth curves of consortia LIG-S and LIG-B on alkali lignin. Both consortia LIG-B and LIG-S were grown in ASW medium with or without 0.4% alkali lignin (AL) at 30°C for 6 days. ASW containing 0.4% AL without inoculation also served as the negative control. Error bars indicate the standard deviation of the mean for sample triplicate (*n* = 3).

To explore the consortia growth with lignin, consortia LIG-B and LIG-S were grown in ASW with alkali lignin (a model compound of lignin, 0.4%, wt/vol) as the sole carbon source. As shown in Fig. 1, consortia LIG-B and LIG-S both showed significant growth during the 6-day incubation, whereas negative controls (consortia grown without alkali lignin or alkali lignin without inoculation) did not, suggesting that both consortia are potential lignin degraders. Particularly, with the almost same initial cell density, the biomass of consortium LIG-B increased more than that of consortium LIG-S under the same culture condition (Fig. 1).

**FIG 2:**
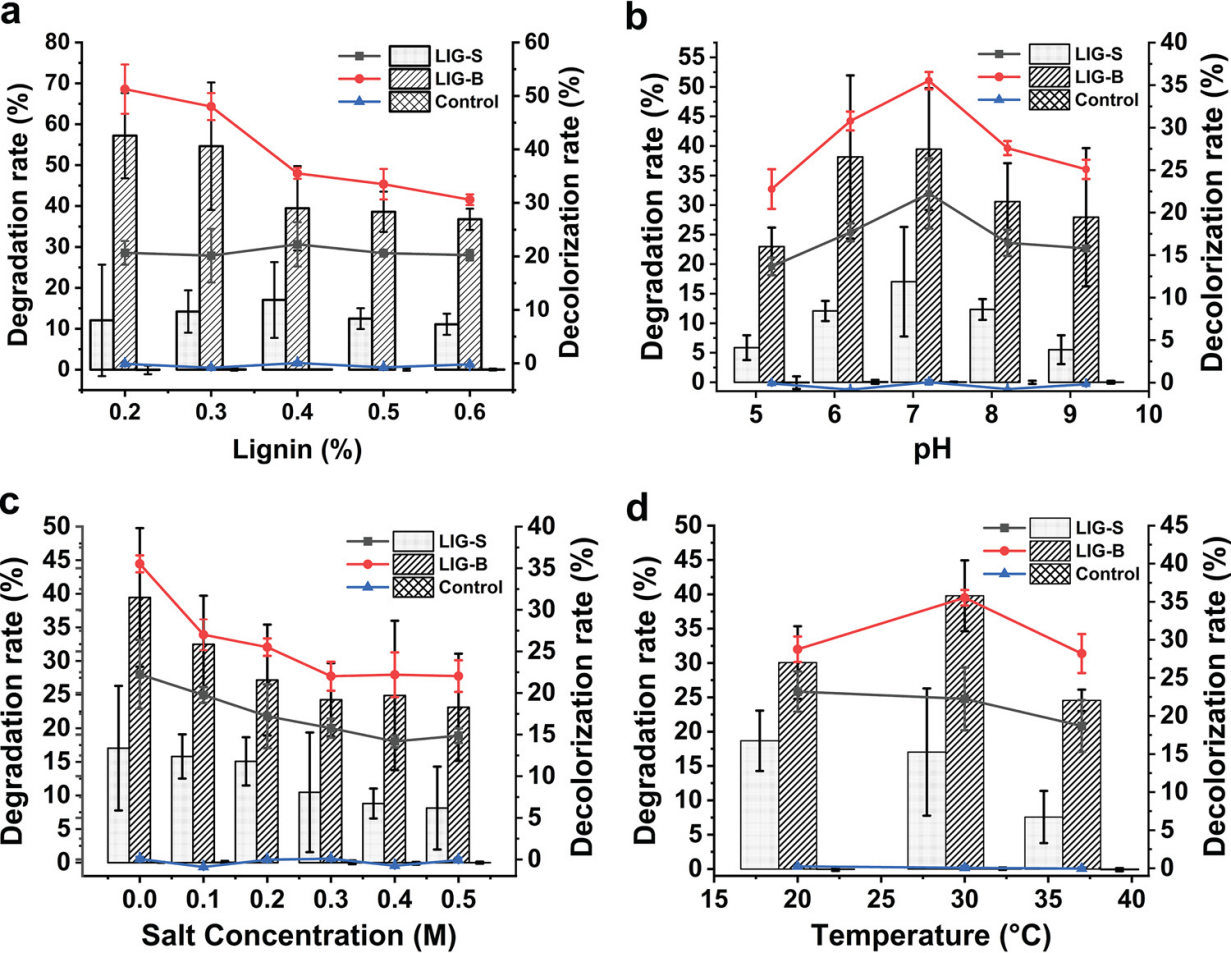
The degradation and decolorization of alkali lignin by consortia LIG-S and LIG-B at different conditions. Both consortia were grown in ASW medium with an incubation of 3 days. LIG-S, samples of consortium LIG-S grown on alkali lignin; LIG-B, samples of consortium LIG-B grown on alkali lignin; Control: alkali lignin without inoculation. (a) Samples of both consortia grown at 30°C and pH 7.2 with 0.2, 0.3, 0.4, 0.5, and 0.6% alkali lignin (wt/vol). (b) Samples of both consortia grown with 0.4% alkali lignin at 30°C and pH values of 5.2, 6.2, 7.2, 8.2, and 9.2. (c) Samples of both consortia grown with 0.4% alkali lignin at 30°C and pH 7.2 with additional NaCl of 0, 0.1, 0.2, 0.3, 0.4, and 0.5 mol L^−1^. (d) Samples of both consortia grown with 0.4% alkali lignin at pH 7.2 and 20°C, 30°C, and 37°C. The bar charts represent the degradation rates (%) while the lines represent the decolorization rates (%). Error bars indicate the standard deviation of the mean for sample triplicate (*n* = 3).

To determine the alkali lignin degradation and its decolorization by each consortium at various conditions, the absorbance at 280 nm and the absorbance at 465 nm (two characteristic absorption peaks for alkali lignin) of the supernatants were both measured. Alkali lignin without inoculation served as the negative control. As shown in Fig. 2, the overall lignin degradation and decolorization efficiencies were more favorable by consortium LIG-B than by consortium LIG-S at all examined conditions, and the lignin decomposition efficiency by each consortium was affected by the culture conditions. Coincidentally, for both consortia, the lignin degradation and decolorization rates followed a similar trend within each treatment group. Based on further statistical analysis, the lignin decomposition abilities between the two consortia were significantly different (*P < *0.05) for each treatment group (Table S5). Meanwhile, the significance of the decomposition abilities was also shown more distinctly in the decolorization analysis, particularly in the case of various lignin concentrations (*P = *0.000). However, the effects of different treatment groups on lignin degradation for the two consortia were not significant (*P > *0.05).

Nonetheless, both consortia were able to efficiently degrade 0.2 to 0.6% alkali lignin (wt/vol) at 20 to 37°C and pH 5.2 to 9.2 in ASW with 0 to 5 mol L^−1^ additional NaCl. In addition, the optimum conditions for the degradation and decolorization by LIG-B in ASW were 0.2% alkali lignin (wt/vol), pH 7.2, 30°C and without additional NaCl. As to LIG-S, the optimum conditions were 0.4% alkali lignin (wt/vol), pH 7.2, 20°C and without additional NaCl. In particular, the maximum degradation rates of lignin by consortia LIG-B and LIG-S were 57% and 18% at their optimum conditions, respectively. Although the maximum degradation rate by consortium LIG-B was higher than that by consortium LIG-S at their respective optimum conditions, it might be premature to claim that consortium LIG-B had a better degradation ability or higher specific activities. However, based on the higher biomass of consortium LIG-B compared with consortium LIG-S, it probably suggested that the more biomass was one of the reasons for the better degradation by consortium LIG-B.

### Morphological changes of lignin after biotreatment.

Morphological changes after biotreatment were also investigated to further characterize lignin degradation by both consortia through SEM imaging. The SEM images of untreated alkali lignin and biotreated alkali lignin are shown in Fig. S2. It was evident that the untreated alkali lignin (Fig. S2a) consists of small spherical fragments, densely aggregated together, whereas, biotreated alkali lignin by each consortium had become more irregular and smaller in size (Fig. S2b and c). Particularly, the LIG-S-treated sample was observed to be highly fragmented with relatively smaller particles (Fig. S2c) compared to LIG-B-treated lignin (Fig. S2b). This was similar to a case of the morphological changes in alkali lignin structure after the treatment by a bacterial strain, Bacillus ligniniphilus L1, previously (32). The morphological changes of lignin after the biotreatment suggested that both consortia can effectively disrupt the lignin structure and might subsequently provide evidence for lignin degradation.

### Intermediates of lignin degradation by bacterial consortia.

Gas chromatography-mass spectrometry (GC-MS) was used to detect and identify intermediates of lignin degradation by consortia LIG-B and LIG-S for 5 days, and alkali lignin medium without bacteria inoculation was the negative control. The total ion current chromatograms are shown in Fig. S3, and the main detected compounds are summarized in Table 1. In the negative control, a number of lignin-derived low molecular-weight aromatics were detected including phenol, guaiacol, 4-hydroxybenzaldehyde, vanillin, 4-hydroxybenzoic acid, vanillate, *m*-coumaric acid, and *p*-coumaric acid (Table 1). The presence of compounds detected in the negative control may be due to the residual by-products of the alkali lignin sample in the manufacturing process (33).

**TABLE 1:** Identification of intermediates during the degradation of alkali lignin by consortia LIG-B and LIG-S using GC-MS^*a*^

No.	RT/min	Samples			Degradation products
		Negative control	LIG-B	LIG-S	
1	5.57	+	+	+	Formic acid
2	6.38	+	+	+	Ethylene glycol
3	7.36	+	−	−	Phenol
4	7.45	+	−	−	Lactic acid
5	7.66	+	−	−	Glycolic acid
6	7.88	+	+	+	2-Ethoxyethanol
7	8.42	+	−	−	2-Hydroxybutyric acid
8	8.55	−	+	+	2-Hydroxyisobutyric acid
9	9.30	+	−	−	Levulinic acid enol
10	9.42	+	−	−	2-Hydroxyvaleric acid
11	9.49	+	−	−	Malonic acid
12	9.83	+	−	−	Guaicol
13	10.81	−	+	−	2-Hydroxyisocaproic acid
14	11.00	+	−	−	Succinic acid
15	11.17	+	−	−	Methyl succinic acid
16	11.87	+	−	−	4-Hydroxybenzaldehyde
17	12.10	−	−	+	2,3-Dimethylsuccinic acid
18	12.14	−	+	−	Malic acid
19	12.41	−	−	+	Methylmalonic acid
20	13.09	−	+	−	Acetophenone
21	13.91	+	−	−	Vanillin
22	14.32	+	−	−	2-Hydroxyglutaric acid
23	14.92	−	+	−	4-Hydroxy-3-methoxyacetophenone
24	15.02	+	−	−	4-Hydroxybenzoic acid
25	15.57	−	+	+	2,6-Dimethoxyhydroquinone
26	15.75	+	−	−	1,2-Benzenedicarboxylic acid
27	15.90	−	+	−	4,5-Dimethoxy-2-[(E)-2-phenylethenyl] benzoic acid
28	16.49	+	−	−	Vanillic acid
29	16.66	+	+	−	Methylhydroquinone
30	16.75	+	−	−	*m*-Coumaric acid
31	17.26	−	+	−	Tetradecanoic acid
32	17.90	+	−	−	4-Hydroxy-3-methoxyphenylpropionic acid
33	18.28	+	−	−	*p*-Coumaric acid
34	19.18	−	−	+	Hexadecanoic acid
35	20.95	+	+	−	Octadecanoic acid

a Negative control, alkali lignin; LIG-B, alkali lignin treated by consortium LIG-B; LIG-S, alkali lignin treated by consortium LIG-S; +, the presence of compounds; −, the absence of compounds.

However, in two biotreated samples, all aforementioned compounds disappeared (Table 1). This indicated both consortia were able to degrade these lignin intermediates by breaking the β-aryl ether (β-O-4) and biphenyl (5-5) linkages, similar to the cases of lignin degradation by terrestrial bacteria and fungi (15, 24). Meanwhile, the appearance of phenylpropane derivates such as 2-hydroxyisobutyric acid (retention time [RT], 8.55 min), acetophenone (RT, 13.09 min), 4-hydroxy-3-methoxyacetophenone (RT, 14.92 min), 2,6-dimethoxyhydroquinone (RT, 15.57 min), and 4,5-dimethoxy-2-[(E)-2-phenylethenyl] benzoic acid (RT, 15.90 min) in LIG-B sample clearly validated the lignin degradation by this consortium (Table 1). Furthermore, the presence of acetophenone and acetophenone derivates found in LIG-B treated sample were also the indicative of β-O-4 bonds cleavage catalyzed by bacterial laccases in consortium LIG-B (34). Similarly, the disappearance of the aforementioned original low molecular-weight aromatics was accompanied by the appearance of new low-molecular-weight compounds (2-hydroxyisobutyric acid, RT, 8.55 min; 2,3-dimethylsuccinic acid, RT, 12.10 min; methylmalonic acid, RT, 12.41 min; hexadecanoic acid, RT, 19.18 min) in LIG-S sample (Table 1). These suggested that both consortia were capable of depolymerizing lignin into small molecular fragments and utilizing them for growth.

### Ligninolytic enzyme activities.

The secretion of extracellular enzymes, such as LiP, MnP, and Lac, is an important component in the biodegradation of lignin polymer via microbes and has been widely characterized in the literature (16, 35, 36). Hence, the activities of these enzymes in both consortia were assayed to identify their ligninolytic function.

**FIG 3:**
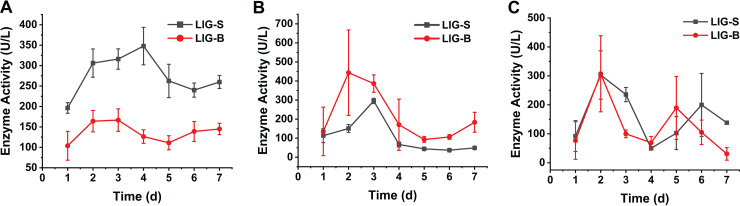
The ligninolytic enzyme activities in consortia LIG-S and LIG-B. (A). Enzyme activity of Lac. (B) Enzyme activity of MnP. (C) Enzyme activity of LiP. The enzymatic assay was performed using the crude enzyme solution. Lac activity was measured using ABTS as the substrate by monitoring the increase of the absorbance of 420 nm (ε_420_ = 36,000 L [mol^−1^ cm^−1^]). MnP and LiP activities were determined by monitoring the increases of the absorbance at 469 nm (ε_469_ = 49,600 L [mol^−1^ cm^−1^]) and 310 nm (ε_310_ = 9,300 L [mol^−1^ cm^−1^]) using 2,6-dimethoxyphenol (2,6-DMP) and veratyl alcohol (VA) as substrates, respectively. All enzymatic assays were performed at pH 3.0 and 30°C. Error bars indicate the standard deviation of the mean for the sample triplicate (*n* = 3).

Both consortia exhibited 2,2′-azino-bis(3-ethylbenzothiazoline-6-sulfonic acid) (ABTS)-oxidizing activity with a similar trend indicating the presence of Lac activities (Fig. 3A). The activity in LIG-S was evidently higher than that in consortium LIG-B. Lac activity increased significantly reaching a maximum activity (348 U L^−1^) on the fourth day for LIG-S whereas the maximum activity for consortium LIG-B (166 U L^−1^) was observed on the third day. However, both showed a slight decrease after reaching maximum activity in the subsequent time (Fig. 3A). Enzyme DyP in the bacterial consortia was reported to act on similar substrates such as ABTS and 2,6-dimethoxyphenol (2,6-DMP) (37, 38), whose enzymatic mechanism was similar to that of LiP and MnP (16). In this study, the DyP encoding gene in the metagenomes of the bacteria consortium LIG-S showed a positive hit, with a sequence identity of 39%. Random binding of DyP and substrate may also occur during the enzymatic assay of DyP in the absence of peroxide (39), suggesting that the higher enzyme activity of LIG-S shown in Fig. 3A could be the result of the synergistic action of two enzymes, Lac and DyP.

MnP activity, on the other hand, was relatively higher in consortium LIG-B compared to that in consortium LIG-S (Fig. 3B). Consortia LIG-B and LIG-S also exhibited a similar trend of MnP activity with a rapid increase until reaching the maximum values on the second and third day, respectively. An approximately 2.3-fold increase, from 135 to 444 U L^−1^, in MnP activity was observed in LIG-B within the first 2 days (Fig. 3B). As for LIG-S, it reached a maximum of 295 U L^−1^ (from the initial activity 135 U L^−1^, approximately 1.2-fold increase) followed by declining activities reaching a plateau of around 40 U L^−1^ at the end of the time course performed (Fig. 3B).

On the contrary, the LiP enzyme assay was of poor resolution where no visible trend can be observed (Fig. 3C). LiP activity for both consortia showed significant fluctuations, which were similar to those reported previously (40). Nonetheless, the formation of veratyl alcohol by LiP was still detected from the enzymatic assay for both consortia with the highest activity at around 300 U L^−1^ on the second day (Fig. 3C).

In this study, the overall lignin degradation efficacy was observed higher in consortium LIG-B than in LIG-S, even with a higher laccase activity present in consortium LIG-S (Fig. 2). Previously, it was reported that lignin peroxidases such as MnP and LiP had higher redox potential compared to laccase allowing them to act on both phenolic and nonphenolic components of lignin (15), resulting in an overall better lignin decomposition by consortium LIG-B. Considering the aforementioned more biomass increase in consortium LIG-B (Fig. 1), it was suggested that the more bacteria numbers and higher specific activities led to the better degradation and decolorization of lignin by consortium LIG-B. Furthermore, both consortia have shown the ability to secrete more than one ligninolytic enzyme, indicating that bacterial consortia are probably more effective compared to other studies using single isolates. For instance, the Lac and MnP activities in this study were found at least 150- and 400-fold higher than those reported in single bacteria isolates, respectively (41, 42). The maximum degradation rates of single bacterial isolates reported in their studies were also lower than those of consortium LIG-B.

### Lignin degradation network analysis from metagenomes.

Based on previous reports, the complete mineralization of lignin consists of two major steps, depolymerization and ring cleavage (22, 43). The prior ligninolytic enzyme identification and GC-MS analysis indicated that the lignin polymer was able to be depolymerized to a mixture of heterogeneous aromatics including monolignols, entering the upper (44) or funneling pathway (45), leading to the formation of vanillin and vanillate. In this study, upon conducting the metagenomic sequencing, 9,138 and 12,367 coding DNA sequences (CDS) were annotated from consortia LIG-B and LIG-S, respectively, some of which consist of lignin degradation genes (Table S1). The number of positive hits of lignin degradation genes involved in the upstream pathway via BLASTp were summarized in Table 2. Based on the metagenomic analysis, the potential lignin degradation network was suggested as follows.

**TABLE 2:** Gene abundance (CDS) of lignin-derived aromatics degradation pathways from the metagenomes of consortia LIG-B and LIG-S

Pathway/Genes	Gene products	Gene abundance (CDS)	
		LIG-B	LIG-S
Benzoate to catechol			
*benA-xylX*	2-Halobenzoate 1,2-dioxygenase large subunit	1	4
*benB-xylY*	2-Halobenzoate 1,2-dioxygenase small subunit	0	4
*benC-xylZ*	Benzoate 1,2-dioxygenase electron transfer component	0	3
*benD-xylL*	Levodione reductase	10	52
Vanillin to protocatechuate			
*vdh*	Vanillin dehydrogenase	20	85
*VanA*	Vanillate monooxygenase oxygenase subunit	0	0
*VanB*	Vanillate *O*-demethylase oxidoreductase	1	6
4HBA to protocatechuate			
*pobA*	*p*-Hydroxybenzoate hydroxylase	1	4
*pobR*	Arabinose operon regulatory protein	1	2
*praI*	4-Hydroxybenzoate 3-hydroxylase	1	4
β-Aryl ether to vanillin			
*ligD*	C alpha-dehydrogenase	5	19
*ligE*	Beta-etherase	1	2
*ligF*	Beta-etherase	0	0
*ligG*	Glutathione *S*-transferase homolog	0	0
*ligP*	Beta-etherase	1	0
*ligQ*	GSH-dependent disulfide-bond oxidoreductase	1	4
Biphenyl to vanillate			
*ligW2*	5-Carboxyvanillic acid decarboxylase	0	1
*ligW*	5-Carboxyvanillate decarboxylase	0	1
*ligX*	DDVA *O*-demethylase	1	1
*ligY*	2,2′,3-Trihydroxy-3′-methoxy-5,5′-dicarboxybiphenyl meta-cleavage compound hydrolase	0	2
*ligZ*	OH-DDVA oxygenase	0	0
Ferulate to vanillin			
*ferA*	Feruloyl-CoA synthetase	0	0
*ferB*	Feruloyl-CoA hydratase/lyase	3	2
*ferB2*	Feruloyl-CoA hydratase/lyase	7	26
*p*-Coumarate to vanillate			
*fcs*	Feruloyl-CoA-synthetase	0	0
*vdh*	Vanillin dehydrogenase	20	85
*ech*	Hydroxycinnamoyl-CoA hydratase-lyase	4	13
Syringate to protocatechuate			
*desA*	Syringate *O*-demethylase	0	4
*desB*	Gallate dioxygenase	0	1
*desZ*	3-*O*-methylgallate 3,4-dioxygenase	0	0
*ligM*	3-*O*-methylgallate *O*-demethylase	0	4

**(i) Upstream degradation.** Monolignols that construct the lignin complex were linked together by different linkages, which include β-O-4, 5-5, β-1, α-1, α-O-4, dibenzodioxins, and 4-O-5 bonds (9). β-Aryl ether (β-O-4) linkages account for approximately 50% of all the ether bonds (9, 46) while biphenyl (5-5) compounds are the most robust C-C bonds present in lignin. Based on genes annotated in *S. paucimobilis* SYK-6 (47), β-aryl ether linkage cleavage is driven by LigE, LigF, LigG, LigP, and LigQ in turn, producing vanillin. On the other hand, biphenyl cleavage is done by LigX, LigY, LigZ, LigW, and LigW2, resulting in the formation of vanillate. In this study, metagenomes analysis revealed that both consortia consist of one or more CDS relevant to the β-aryl ether pathway but were incomplete for the biphenyl pathway (Table 2). Thus, it indicated that both consortia were unable to thoroughly utilize lignin-derived biphenyl compounds.

Genes involved in degrading other more common model lignin derivative compounds such as syringate and vanillin, and secondary intermediates such as benzoate were also identified in the metagenomes of consortium LIG-S but not in consortium LIG-B (Table 2). The conversion of vanillin to vanillate is done by vanillin dehydrogenase encoded by a *vdh* gene, followed by either VanAB or LigM to subsequently convert vanillate to protocatechuate (PCA), a ring-cleavage substrate. Both consortia contained the genes involved in the conversion of vanillin to PCA. However, the frequencies of the positive CDS encoding the above conversion in consortium LIG-S were also relatively higher than those in consortium LIG-B.

In previous reports, *p-*hydroxycinnamates such as ferulate and *p*-coumarate are other important intermediates that were converted to vanillin via enzyme encoded by the (i) *fcs* and *ech* genes (48) and (ii) *ferAB* genes (49) or to vanillate encoded by (iii) *couNOM* genes (50). In this study, a metagenomic search failed to find the gene encoding the initial step, *ferA*/*fcs* from both metagenomes of these two consortia. However, the genes in the subsequent step (*ferB*, *ferB2*, and *ech*) were present. The absence of the initial CDS indicated that these compounds were unable to form vanillin as intermediate compounds, although the GC-MS chromatogram showed that ferulic acid and *p*-coumaric acid were metabolized. This might be due to the vast diversity of the bacteria species in the metagenome compared to the origin of the query protein sequenced used, showing very low sequence identity or none at all. The absence of initial genes (*ferA* and *fcs*) being responsible for the addition of CoA molecules leads to the speculation of marine consortia harboring genes encoding a possible phenolic acid decarboxylase and undergoing a nonoxidative decarboxylation pathway (19) and forming vinyl compounds such as 4-vinyl phenol and 4-vinyl guaiacol from *p*-coumaric acid and ferulic acid, respectively (23). This pathway was also found in many bacteria, including those of marine lineages (23). Thus, it suggested that consortia LIG-B and LIG-S probably undergo nonoxidative carboxylation for ferulic and *p*-coumaric acid catabolism.

**(ii) Central metabolite degradation.** Most lignin-derived aromatic compounds form central intermediates such as catechol and PCA, which enter the β-ketoadipate pathway (18–20) and finally into the tricarboxylic acid (TCA) cycle (45). PCA degradation can be divided into three degradation pathways, namely, (i) PCA 2,3-cleavage pathway (18), (ii) PCA 3,4-cleavage pathway, and (iii) PCA 4,5-cleavage pathway which eventually forms acetyl-CoA, pyruvate or succinate that enters the TCA cycle (44). As for the catechol degradation, it consists of catechol 1,2-cleavage pathway and catechol 2,3-cleavage pathway.

**FIG 4:**
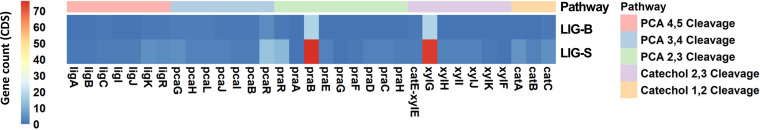
Gene abundance of catabolic genes involved in the ring cleavage of the lignin-derived aromatic compounds. The gradient bar on the left exhibits the gene count (CDS) in both metagenomes, whereas the bars on top represent the different PCA and catechol ring cleavage routes identified from each metagenome. The descriptions of all genes and their encoded products are listed in Table S4.

In this study, the genes involved in the different branches of the β-ketoadipate pathway via catechol and PCA were annotated in the metagenomes of consortia LIG-B and LIG-S. Furthermore, the abundance of genes involved in the different ring cleavages of PCA and catechol was analyzed to determine which pathway marine bacteria undergo. As shown in Fig. 4, the abundance of genes in the multiple central metabolite pathways was higher in consortium LIG-S than in consortium LIG-B. The metagenome present in consortium LIG-S showed more genetic versatility compared to that in consortium LIG-B in regards to lignin degradation. In addition, the metagenomic analysis also showed that consortium LIG-B possibly decomposes lignin-derived compounds through PCA 3,4-cleavage (*ortho*) pathway only, while LIG-S mineralizes the lignin-derived compounds via multiple central metabolite pathways. The high abundance of numerous lignin degradation genes (Fig. 4) also suggested a high degree of functional redundancy as multiple bacteria in both consortia were able to carry out the same reaction.

**FIG 5:**
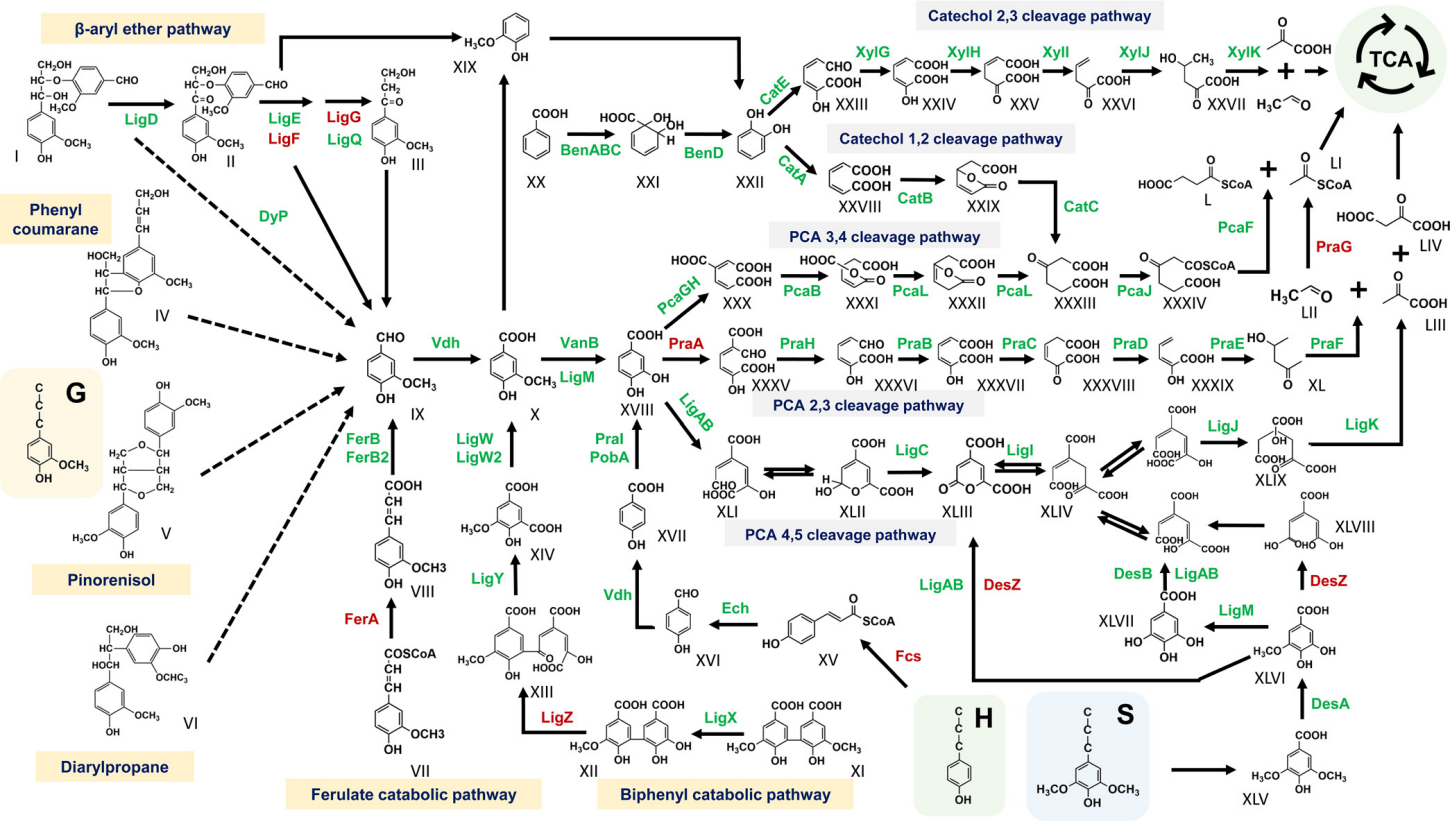
The reconstructed lignin degradation network map for consortium LIG-S based on the presence of genes in the metagenome. Green and red labels indicated functional proteins encoded by the presence and absence of genes involved in each pathway in the metagenome of consortium LIG-S, respectively. Dotted arrows indicate the degradation routes that remain elusive. The descriptions of all genes and their encoded products are listed in Table S4. Compounds: I, β-aryl ether; II, α-(2-methoxyphenoxy)-β-hydroxypropiovanillone; III, β-hydroxypropiovanillone; IV, phenyl coumarane; V, pinoresinol; VI, siarylpropane; VII, ferulate; VIII, feruloyl-CoA; IX, vanillin; X, vanillate; XI, 5,5′-dehydrodivanillate (DDVA); XII, 2,2′,3-trihydroxy-3′-methoxy-5,5′-dicarboxybiphenyl (OH-DDVA); XIII, OH-DDVA meta-cleavage product; XIV, 5-carboxyvanillic acid; XV, coumaroyl-CoA; XVI, 4-hydroxybenzaldehyde; XVII, 4-hydroxybenzoate; XVIII, protocatechuate; XIX, guaiacol; XX, benzoate; XXI, *cis*-1,2-dihydroxycyclohexa-3,5-diene-1-carboxylate; XXII, catechol; XXIII, 2-hydroxymuconate semialdehyde; XXIV, 2-hydroxy-muconate; XXV, 4-oxalocrotonate; XXVI, 2-oxopent-4-enoate; XXVII, 4-hydroxy-2-oxopentanoate; XXVIII, *cis*,*cis*-muconate; XXIX, muconolactone; XXX, 3-carboxy-*cis*,*cis*-muconate; XXXI, 4-carboxymuconolactone; XXXII, 3-oxoadipate enol-lactone; XXXIII, 3-oxoadipate; XXXIV, 3-oxoadipyl-CoA; XXXV, 5-carboxy-2-hydroxymuconate-6-semialdehyde; XXXVI, 2-hydroxymuconate-6-semialdehyde; XXXVII, 2-hydroxymuconate; XXXVIII, 4-oxalocrotonate; XXXIX, 2-hydroxypenta-2,4-dienoate; XL, 4-hydroxy-2-oxovalerate; XLI-XLII, 4-carboxy-2-hydroxymuconate semialdehyde; XLIII, 2-pyrone-4,6-dicarboxylate; XLIV, 4-oxalomesaconate; XLV, syringate; XLVI, 3-*O*-methylgallate; XLVII, gallate; XLVIII, 4-carboxy-2-hydroxy-6-methoxy-6-oxohexa-2,4-dienoate; XLIX, 4-carboxy-4-hydroxy-2-oxoadipate; L, succinyl-CoA; LI, acetyl-CoA; LII, acetylaldehyde; LIII, pyruvate; LIV, oxaloacetate.

Furthermore, 13 suggested pathways included in the degradation network are listed in Table S2. The results showed that more than half of the pathways (9 pathways) were complete in consortium LIG-S, whereas only 4 pathways were complete in consortium LIG-B. Overall, the metagenomes of consortium LIG-S reflected a more comprehensive degradation network according to the CDS identity. Based on these, a lignin degradation network in consortium LIG-S was reconstructed and is illustrated in Fig. 5.

In summary, the metagenomic analysis of consortia LIG-B and LIG-S covers the majority of the metabolic pathway related to the lignin-derived aromatic compounds degradation discovered, especially those genes were found in the lignin-degrader S. paucimobilis SYK-6 (24), *R. jostii* RHA1 (25, 26), and P. putida mt-2 (25, 51). At the genomic level, the lignin degradation system of consortium LIG-S exhibited a higher integrity compared to consortium LIG-B. The reconstructed degradation network in this study provides a better understanding of lignin degradation driven by bacteria in the marine environment. On the other hand, ring cleavage is an important component in aromatic compound degradation, especially in lignin polymer that is comprised of complex polyaromatic subunits. Metagenomic analysis of consortia LIG-B and LIG-S shows multiple upper or funneling pathways of lignin model compound degradation ultimately leading to the formation of PCA and catechol. Among the three possible PCA pathways mentioned above, PCA 2,3 cleavage route was incomplete in both consortia here, which was echoed by the fact that PCA 2,3-dioxygenase activity was not as commonly found in both marine and terrestrial microorganisms (18). Previous studies have shown a marine bacterium Pseudomonas aeruginosa JP-11 consisting of a catechol 1,2-dioxygenase pathway (52) and several marine bacteria exhibiting PCA 3,4-dioxygenase and PCA 4,5-dioxygenase activity for the PCA ring cleavage (53), which is consistent with our metagenomic analysis here.

### Distribution and abundance of lignin-related degradative genes in global similar nearshore regions.

**FIG 6:**
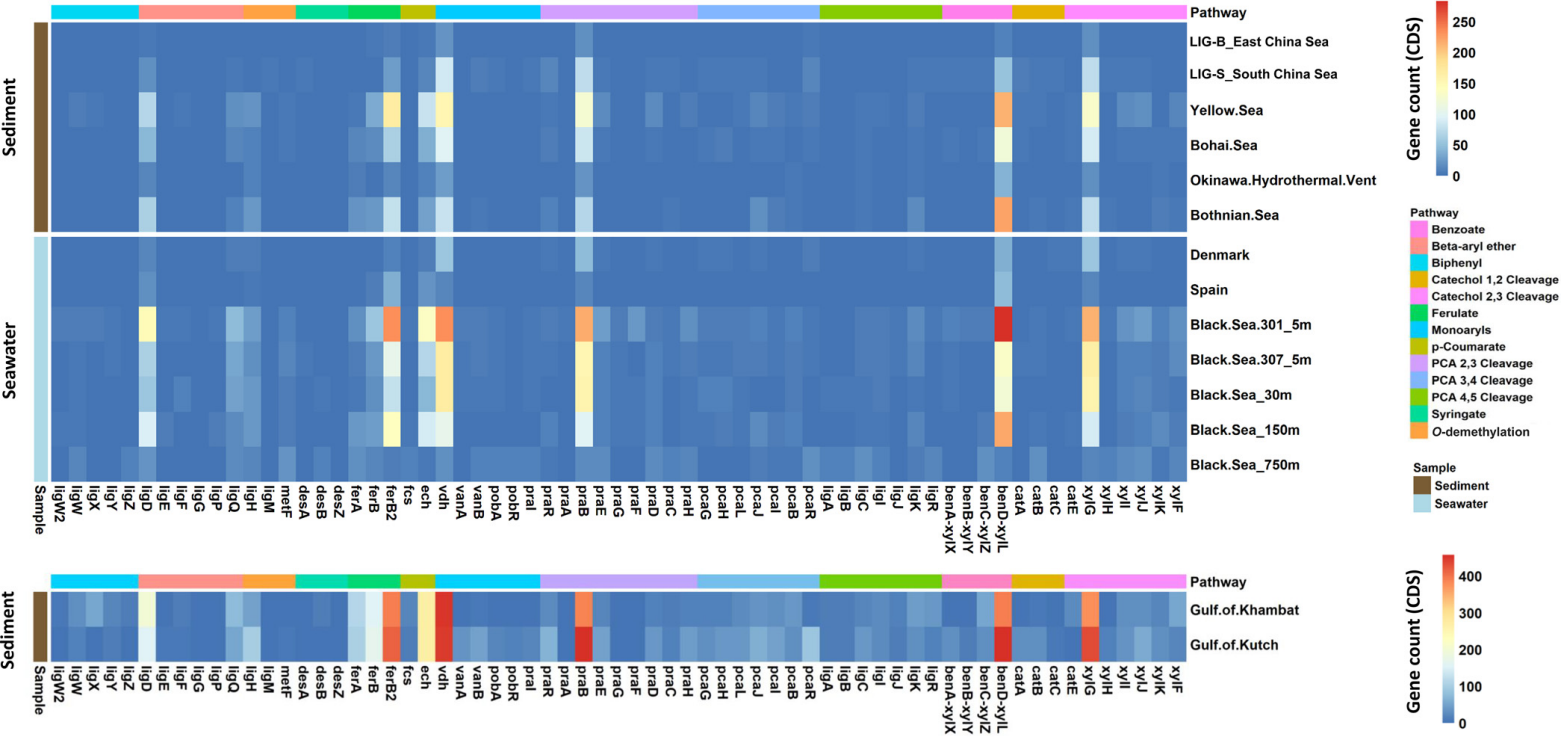
Gene abundance of catabolic genes encoding the ring cleavage of lignin-derived aromatics in 783 publicly available metagenome-assembled genomes derived from nine nearshore regions and two metagenomes of consortia LIG-B and LIG-S. The bars on the left represent the type of each metagenomic sample whereas the top bars with different colors indicate the different types of pathways. The description of all genes and their encoded products are listed in Table S4.

To explore the global lignin degradation driven by marine bacteria in nearshore regions, the distribution and abundance of catabolic genes involved in the degradation of lignin and its derivatives were also analyzed in 783 publicly available metagenomic-assembled genomes (MAGs) of nine similar nearshore regions and two metagenomes of consortia LIG-B and LIG-S in this study (Table S3). As shown in Fig. 6, there were no distinctive gene abundance patterns observed in the various locations compared to those in the East and South China Seas. Notably, the results for the Gulf of Khambat and Gulf of Kutch in the Arabian Sea were separated due to their large sample size and numerous gene count (CDS) identified from their metagenomes compared to those in other regions analyzed. The large sample size and numerous CDS of the above two particular regions were mostly derived from the high abundance of lignin organic matter from the covering forest reserves along the coastal line.

Meanwhile, the genes involved in the ferulate pathway and benzoate-catechol pathway show a significantly higher abundance compared to the other pathways in the lignin degradation, indicating that these might be the major pathways by which lignin-derived aromatic compounds underwent (Fig. 6). Moreover, LigD involved in the initial stage of β-aryl ether compound catabolism and *vdh* genes involved in the conversion of vanillin to vanillate were also relatively higher in abundance in the available nearshore regions metagenomes compared to the other lignin-related catabolic genes, which is consistent with the results observed in the East and South China Seas in this study. Most regions shared a similar gene abundance trend indicating that the marine bacteria in global similar nearshore regions undergo the similar degradation pathways as consortia LIG-B and LIG-S to drive lignin degradation as part of the oceanic carbon cycle, regardless of in sediments or in water column.

Furthermore, a similar study conducted in an aquatic system also reported the positive involvement of Proteobacteria with identical degradation pathways as in this study (28). Combining our research with the previous report (28), it was suggested that bacteria possess similar strategies for lignin degradation both in the freshwater system and the shallow marine environment.

### Conclusion.

Lignin is the second most abundant biopolymer found in vascular plants, and the constant discharge of lignin into the ocean should display a higher concentration of lignin in the marine system. However, the amount of lignin detected is relatively low compared to the sedimentation rate. In this study, two bacterial consortia enriched from the nearshore regions of the ocean have shown the ability to utilize alkali lignin (a model compound of lignin) for their growth. Significant degradation and decolorization of alkali lignin occurred with the increasing biomass. The newly emerged low-molecular-weight compounds in biotreated alkali lignin also evidently showed the depolymerization of lignin by both consortia. Furthermore, both consortia could secret multiple ligninolytic enzymes (Lac, MnP, and LiP) similar to those found from terrestrial bacteria and fungi. The lignin degradation pathways were reconstructed from consortium LIG-S, and the catabolic genes were present not only in consortia LIG-B and LIG-S but also in 783 publicly available metagenomic-assembled genomes from nine nearshore regions. This suggests that marine microorganisms, particularly the nearshore-region-bacteria, undergo similar degradation mechanisms as terrestrial organisms via depolymerization, aromatic degradation, ring cleavage, and ultimately integrated into the TCA cycle. Moreover, the lignin degradation pathways reconstructed here showed the complete capability of lignin mineralization in the marine system, indicating the incorporation of lignin in the oceanic carbon cycle. However, further research is still required to understand the role of dominant bacteria species and how each of them contributes to native lignin degradation in the marine environment.

## MATERIALS AND METHODS

### Chemicals and growth medium.

Alkali lignin was purchased from Adamas-beta, and ASW used in this study contained 24 g L^−1^ NaCl, 1 g L^−1^ NH_4_Cl, 0.7 g L^−1^ KCl, 2 g L^−1^ KH_2_PO_4_, and 3 g L^−1^ Na_2_HPO_4_·12H_2_O, pH 7.2 to 7.4, along with the addition of 2% trace element containing 10 g L^−1^ MgSO_4_·7H_2_O, 1 g L^−1^ CuSO_4_·5H_2_O, 1 g L^−1^ FeSO_4_·7H_2_O, 1 g L^−1^ CaCl_2_, and 1 g L^−1^ MnSO_4_.

### Screening and enrichment of consortia.

Two marine sediment samples were collected from the surface of the sedimentary layer (about 200 m below sea level) in the nearshore regions of the East Sea (122.0079° E, 30.0155° N) and the South China Sea (113.9124° E, 22.0362° N), respectively. In addition, they were kept in the dark at −4°C under a sealed condition until use. One gram of each sediment sample was suspended separately in 100 mL of sterile water, and 1 mL of each sample was then transferred into a 100 mL ASW with 0.4% alkali lignin (wt/vol) as the sole carbon source. The culture media were incubated at 30°C and 150 rpm for 72 h. The enrichments with ASW were repeated for five rounds before the lignin degradation consortia were isolated. The two lignin degradation consortia from the East China Sea and South China were named LIG-B and LIG-S, respectively, which were stored at −80°C in 25% glycerol for further use.

### Growth of lignin degradation consortia.

Consortia LIG-B and LIG-S were inoculated into fresh ASW with 0.4% alkali lignin (wt/vol) and maintained in a shaking incubator at 30°C and 150 rpm without the addition of any gas. Samples were then taken at every 24-h time interval for 6 days to measure bacterial growth. The samples were subjected to centrifugation at 8,000 × *g* for 5 min followed by suspension in phosphate buffer of pH 7.4. Bacterial cells from each consortium were washed three times with the buffer followed by resuspension in 1 mL of ASW. The growth of the culture was evaluated by measuring the absorbance at an optical density at 600 nm (OD_600_) via a spectrometer (Lambda 25 UV/VIS; PerkinElmer).

### Lignin degradation and decolorization analysis.

The efficacy of lignin degradation and decolorization of each consortium was evaluated at different conditions with 7 days of incubation: (i) both consortia grown in ASW at 30°C and pH 7.2 with 0.2, 0.3, 0.4, 0.5, and 0.6% alkali lignin (wt/vol); (ii) both consortia grown in ASW with 0.4% alkali lignin at 30°C and pH values of 5.2, 6.2, 7.2, 8.2, and 9.2; (iii) both consortia grown in ASW with 0.4% alkali lignin at 30°C and pH 7.2 with additional NaCl of 0, 0.1, 0.2, 0.3, 0.4, and 0.5 mol L^−1^; and (iv) both consortia grown in ASW with 0.4% alkali lignin at pH 7.2 and 20°C, 30°C, and 37°C.

Standard procedures in measuring lignin degradation and decolorization rate were previously reported (54, 55). The decrease in absorbance at 280 nm implied the decomposition of lignin molecule (54), and the degradation rate was defined as the ratio of decrease in lignin concentration before and after bacterial samples were inoculated. The changes in absorbance at 465 nm demonstrate the decolorization of lignin (55). Briefly, LIG-B and LIG-S were inoculated separately into a fresh ASW medium with alkali lignin as a carbon source for 72 h, and an uninoculated sample was used as a negative control. One milliliter of the culture was harvested by centrifugation at 10,000 × *g* for 10 min to remove the lignin biomass. The supernatant was diluted with Na_2_HPO_4_-NaH_2_PO_4_ buffer and the absorbance was measured at OD_280_ and OD_465_ to determine the lignin concentration and decolorization rate, respectively. The absorbance was converted to color units (CU) according to the following calculation: CU = 500 × A_2_/A_1_, where A_1_ indicates the OD_465_ of 500 CU platinum-cobalt standard solution (0.132) and A_2_ refers to the absorbance of samples. The decolorization rate was defined as the ratio of decrease in CU before and after bacterial samples were inoculated. All measurements were performed in triplicates.

### Data analyses.

All statistical analyses were done by using Statistical Package for the Social Sciences (SPSS) ver. 23 (IBM, USA). Data sets were analyzed using the Shapiro-Wilk normality test and were found to follow normal distributions. The data sets were then subjected to multivariate analysis of variance (multivariate ANOVA) parametric tests to analyze for significant differences (α < 0.05). The effects of degradation and decolorization between two consortia and four treatment groups (lignin concentrations, pH values, salt concentrations, and temperatures) were compared.

### Scanning electron microscopy analysis.

Lignin degradation via bacterial consortia was further characterized using scanning electron microscopy (SEM) to observe the surface of lignin. Consortia LIG-B and LIG-S were inoculated in ASW with 0.4% lignin for 7 days in a 150 rpm shaking incubator at 30°C. The culture media were then centrifuged at 10,000 × *g* for 10 min to remove bacterial cells. For SEM observation, the supernatant was subjected to freeze-dried lyophilization and coated with gold-palladium alloy (32).

### Enzyme assays.

Consortia LIG-B and LIG-S were inoculated in ASW with 0.4% alkali lignin (wt/vol) and an uninoculated sample was used as a negative control. The cultures were maintained in a 150 rpm shaking incubator at 30°C. The control and treatment samples were taken at every 24-h interval and centrifuged at 10,000 × *g* for 5 min to obtain the supernatant as a crude enzyme solution. The enzymatic assay using the crude enzyme solution for measuring the ligninolytic enzyme activity was modified based on references 41 and 56 for laccase (Lac) and reference 57 for manganese peroxidase (MnP) and lignin peroxidase (LiP).

Briefly, Lac activity was measured using ABTS as the substrate by monitoring the increase in the absorbance of 420 nm (ε_420_ = 36,000 L [mol^−1^ cm^−1^]) as previously reported (56). Using 2,6-DMP and veratyl alcohol (VA) as substrates, MnP and LiP activities were determined by monitoring the increases of the absorbance of 469 nm (ε_469_ = 49,600 L [mol^−1^ cm^−1^]) and 310 nm (ε_310_ = 9,300 L [mol^−1^ cm^−1^]) as reported previously with the addition of 1 mmol L^−1^ MnSO_4_, respectively, which were both initiated with the addition of 1 mmol L^−1^ H_2_O_2_ (57). One unit of activity (U) is defined as the amount of enzyme that oxidizes 1 μmol of substrate per minute and is presented as U L^−1^. All enzymatic assay was performed at pH 3.0 and 30°C.

### Gas chromatography-mass spectrometry analysis.

GC-MS was performed to characterize the degradation products of alkali lignin. Fifty milliliters of ASW medium with 0.4% alkali lignin (wt/vol) inoculated with consortia LIG-B and LIG-S was incubated for 5 days in a shaking incubator at 30°C. An uninoculated medium was used as the negative control. The cultures were centrifuged at 10,000 × *g* for 10 min. The supernatant was acidified with HCl to pH 1 to 2 followed by the addition of three volumes of ethyl acetate. The organic layer was collected and concentrated to 1 mL using a vacuum rotary evaporator at 40°C. Anhydrous Na_2_SO_4_ was added to remove any remaining water molecules (42, 58). Extracts were then filtered through Whatman No. 54 filter paper and dissolved in 100 μL of dioxane and 10 μL pyridine. The samples were derivatized with 50 μL trimethylsilyl [*N*,*O*-bis(trimethylsilyl)trifluoroacetamide (BSTFA) trimethylchlorosilane (TMCS)]. The mixture was heated at 60°C for 15 min with periodic shaking (42).

An aliquot of 1 μL was injected into the GC-MS (Agilent Technologies, Inc., USA) system equipped with an HP-5MS capillary column (30 m × 0.25 mm × 0.25 μm). Helium was used as a carrier gas with a flow rate of 1 mL min^−1^. The column was held at 60°C for 2 min and increased to 300°C by 10°C min^−1^ and held for 10 min. The transfer line and ion source temperatures were maintained at 200°C and 250°C, respectively. In the full-scan mode, electron ionization mass spectra were recorded at 30 to 500 (*m/z*) at an electron energy of 70 eV. The identification of low-molecular-weight compounds from microbial treatment was analyzed by comparing mass spectra from the National Institute of Standards and Technology 2014 version (NIST 14) library available in the instrument and comparing the retention time (RT) with those of purchased standards.

### Bacteria composition and relative abundance analysis.

Metagenomic sequencing was used to characterize the community structure and bacterial diversity of consortia LIG-B and LIG-S. The consortia were inoculated into ASW with 0.4% alkali lignin maintained in a 150 rpm shaking incubator for 5 days, followed by centrifugation at 8,000 × *g* for 10 min to harvest the cells. The cells were then freeze dried with liquid nitrogen. Both samples were sent to GENEWIZ (Shanghai, China) for metagenomic sequencing. Standard protocols of DNA extraction were performed using the Magen HiPure Soil DNA kit.

Metagenomic shotgun sequencing of the extracted DNA was performed on the Illumina HiSeq platform with 2 × 150-bp paired-end reads providing 10 Gb of output for each sample. Briefly, 100 ng of genomic DNA was randomly fragmentized to <500 bp by sonication (Covaris S220). End repairs of the fragmented DNA were performed using End Prep Enzyme Mix, and fragment sizes of around 470 bp were then recovered using VAHTS DNA Clean Beads. The samples underwent eight cycles of PCR amplification and the PCR products were purified using VAHTS DNA Clean Beads. Lastly, each sample library was validated using an Agilent 2100 Bioanalyzer and quantified by Qubit 3.0 Fluorometer for quality control and quantification purposes.

Samples were then individually assembled using MEGAHIT (v 1.1.3), and contigs were annotated using Prodigal (v 3.02) for open reading frame prediction. Additional annotation of genes was done by comparing against the NCBI NR, eggCOG, KEGG, and CAZy databases. The microbial composition of each consortium was explored using the assembled gene sequences, blasted against the constructed microbial database of bacteria, fungi, archaea, and viruses obtained from the NCBI NT database. The abundance of a species in each sample was denoted by the sum of the gene abundance annotated for the species. All metagenomic data sets from this work were deposited to the Sequence Read Archive (SRA) database with their accession numbers.

### The abundance of lignin degradation genes.

To detect the abundance of bacterial lignin degradation genes, the lignin-related genes were searched from the metagenomes via BLASTp with the following selection criteria: identity ≥30%, coverage ≥50%, and E value ≤10^−5^. Amino acid sequences for catechol 1,2 dioxygenase (CatA), catechol 2,3 dioxygenase (CatE), protocatechuate 4,5 dioxygenase alpha subunit (LigA), protocatechuate 3,4 dioxygenase (PcaG), protocatechuate 2,3 dioxygenase (PraH), and other related enzymes (Table S4) were retrieved from the NCBI database and used as queries to search for orthologs within the whole-metagenome proteome. Genes involved in reconstructed lignin degradation pathways from a lignin-degrader, *S. paucimobilis* SYK-6 (24), were also retrieved (Table S4). The gene count (CDS) of each lignin degradation gene was summarized to generate a matrix of the relative abundance of each gene. This matrix was then clustered and visualized using the pheatmap R package.

### Microbial metagenomes search.

A total of 783 publicly available metagenome-assembled genomes (MAGs) derived from the marine environment in 9 different locations were retrieved from the NCBI database. The majority of these MAGs were sampled from the nearshore locations with water depths ranging from 0 to 200 m including both seawater and sediment samples (Table S3). The abundance of lignin genes in consortia LIG-B and LIG-S was compared to those in these locations. The gene count (CDS) of each lignin degradation gene was summarized to generate a matrix of the relative abundance of each gene. This matrix was visualized using the pheatmap R package.

### Data availability.

The metagenomic data sets for consortia LIG-B and LIG-S from this work were deposited in the SRA database under SRA accession numbers SRR19975620 and SRR19975619. SRA accession numbers and BioProject numbers of all mentioned metagenomes have also been given in the text.
