# Supplementary material for: Characterization of Two Marine Lignin-Degrading Consortia and the Potential Microbial Lignin Degradation Network in Nearshore Regions
Source: Microbiol Spectr. 2023 Apr 12;11(3):e04424-22. doi: 10.1128/spectrum.04424-22 (PMC10269927; doi:10.1128/spectrum.04424-22)
Supplement: Supplemental file 1 — Tables S1 to S5 and Fig. S1 to S3. Download spectrum.04424-22-s0001.pdf, PDF file, 0.5 MB [file spectrum.04424-22-s0001.pdf]

Supplementary materials

Characterization of two marine lignin-degrading consortia and the potential microbial lignin degradation network in nearshore regions

Yvette Ley [1], Xiao-Yu Cheng [1], Zhi-Yue Ying [1], Ning-Yi Zhou [1], Ying Xu [1,*]

[1] State Key Laboratory of Microbial Metabolism, Joint International Research Laboratory of Metabolic & Developmental Sciences, and School of Life Sciences & Biotechnology, Shanghai Jiao Tong University, Shanghai, China

* Correspondence: Ying Xu. E-mail: ying.xu@sjtu.edu.cn; Tel. & Fax: +86-21-34208261; Mailing address: 800 Dongchuan Road, Shanghai, Shanghai Jiao Tong University, Shanghai, China, 200240.

Keywords: lignin biodegradation, lignin-derived aromatics, ligninolytic enzymes, marine consortia, nearshore regions

Table S1. Metagenomic statistics of the two metagenome sequences of the lignin-degrading consortia LIG-B and LIG-S.

Sample ID	Total sequences	Total bases (bp)	Average length (bp)	N50 (bp)	GC content (%)
LIG-B	9138	5791281	633.76	951	59.49
LIG-S	12367	23581565	1906.81	6593	62.05

Table S2. Degradation pathways in the lignin-degrading consortia LIG-B and LIG-S.

Lignin type	Degradation network	LIG-B	LIG-S
	Upstream degradation		
G	β-aryl ether to Vanillin	+	+
G	Biphenyl to Vanillate	-	-
G	Ferulate to Vanillin	-	-
H	*p*-coumarate to Vanillate	-	-
S	Syringate to Protocatechuate	-	+
	Benzoate to Catechol	-	+
NA	Vanillin to Protocatechuate	+	+
	4-HBA to Protocatechuate	+	+
	Central metabolites degradation		
	Catechol ortho (1,2 Cleavage)	-	+
	Catechol meta (2,3 Cleavage)	-	+
NA	PCA ortho (3,4 cleavage)	+	+
	PCA meta (2,3 Cleavage)	-	-
	PCA meta (4,5 Cleavage)	-	+

Note: G, Guaiacyl; S, Syringyl; H, *p*-hydroxyphenyl; NA, not applicable; +, the genes encoding the degradation pathway are complete; -, the genes encoding the degradation pathway are incomplete.

Table S3. Details of the MAGs retrieved from the NCBI database.

Bioproject ID	Location	No. of MAGs	Depth (m)	Sample type	Reference
PRJNA428417	Yellow Sea	38	73	Sediments	(1)
PRJNA514927	Bohai Sea	15	25	Sediments	(1)
PRJNA514953	Okinawa hydrothermal	25	1550	Sediments	(1)
PRJNA511814	Bothnian Sea	64	215	Sediments	(2)
PRJNA598413	Gulf of Khambhat	132	22	Sediments	(3)
PRJNA598416	Gulf of Kutch	138	35	Sediments	(3)
PRJNA257723	Alcante, Spain	4	75	Seawater	(4)
PRJNA613817	Hellerup harbor, Denmark	8	0.1	Seawater	(5)
PRJNA638805	Black Sea	359	5-750	Seawater	(6)

Table S4. The genes involved in the lignin-related degradation pathway and their respective gene products.

Genes	EC no.	Gene Product
benA-xylX	EC: 1.14.12.10	2-Halobenzoate 1,2-dioxygenase large subunit
benB-xylY	EC: 1.14.12.10	2-Halobenzoate 1,2-dioxygenase small subunit
benC-xylZ	EC: 1.14.12.10	Benzoate 1,2-dioxygenase electron transfer component
benD-xylL	EC: 1.3.1.25	Levodione reductase
vdh	EC: 1.2.1.67	Vanillin dehydrogenase
VanA	EC: 1.14.13.82	Vanillate monooxygenase oxygenase subunit
VanB	EC: 1.14.13.83	Vanillate O-demethylase oxidoreductase
pobA	EC: 1.14.13.2	*p*-Hydroxybenzoate hydroxylase

pobR	NA	Arabinose operon regulatory protein
praI	EC: 1.14.13.2	4-Hydroxybenzoate 3-hydroxylase
ligD	NA	C alpha-dehydrogenase
ligE	NA	Beta-etherase
ligF	NA	Beta-etherase
ligG	EC: 2.5.1.18	Glutathione S-transferase homolog
ligP	NA	Beta-etherase
ligQ	NA	GSH-dependent disulfide-bond oxidoreductase
ligW2	NA	5-Carboxyvanillate decarboxylase
ligW	NA	5-Carboxyvanillate decarboxylase
ligX	NA	DDVA O-demethylase
ligY	NA	2,2',3-Trihydroxy-3'-methoxy-5,5'-dicarboxybiphenyl *meta*-cleavage compound hydrolase
ligZ	NA	OH-DDVA oxygenase
ferA	EC: 6.2.1.34	Feruloyl-CoA synthetase
ferB	EC: 4.1.2.61	Feruloyl-CoA hydratase/lyase
ferB2	EC: 4.1.2.61	Feruloyl-Coa hydratase/lyase
fcs	EC: 6.2.1.34	Feruloyl-CoA-synthetase
ech	EC: 4.1.2.61	Hydroxycinnamoyl-CoA hydratase-lyase
desA	NA	Syringate O-demethylase
desB	EC: 1.13.11.57	Gallate dioxygenase
desZ	NA	3-O-methylgallate 3,4-dioxygenase
metF	EC: 1.5.1.54	5,10-Methylenetetrahydrofolate reductase
catE / xylE	EC: 1.13.11.2	Catechol 2,3-dioxygenase
xylG /praB	EC: 1.2.1.85	2-Hydroxymuconate-6-semialdehyde dehydrogenase
xylH / praC	EC: 5.3.2.6	2-hydroxymuconate tautomerase
xylI	EC: 4.1.1.77	2-oxo-3-hexenedioate decarboxylase
xylF	EC: 3.7.1.9	2-Hydroxymuconic semialdehyde hydrolase
xylJ	EC: 4.2.1.80	2-Oxopent-4-enoate hydratase
xylK	EC: 4.1.3.39	4-Hydroxy-2-oxovalerate aldolase

catA	EC: 1.13.11.1	Catechol 1,2-dioxygenase
catB	EC: 5.3.3.4	Muconolactone delta-isomerase
catC	EC: 5.5.1.1	Muconate cycloisomerase 1
pcaG	EC: 1.13.11.3	Protocatechuate 3,4-dioxygenase alpha chain
pcaH	EC: 1.13.11.3	Protocatechuate 3,4-dioxygenase beta chain
pcaL	EC: 3.1.1.24	3-Oxoadipate enol-lactonase
pcaJ	EC: 2.8.3.5	3-Oxoadipate CoA-transferase subunit B
pcaI	EC: 2.8.3.6	3-Oxoadipate CoA-transferase subunit A
pcaB	EC: 5.5.1.2	3-Carboxy-*cis,cis*-muconate cycloisomerase
pcaR	NA	*pca* regulon regulatory protein
praR	NA	Transcriptional regulator IclR family
praA	EC: 1.13.11.15	Protocatechuate 2,3-dioxygenase
praB	EC: 1.2.1.85	2-Hydroxymuconate-6-semialdehyde dehydrogenase
praE	NA	2-Hydroxypenta-2,4-dienoate hydratase
praG	NA	Acetaldehyde dehydrogenase
praF	EC: 4.1.3.39	4-Hydroxy-2-oxovalerate aldolase
praD	EC: 4.1.1.77	4-Oxalocrotonate decarboxylase
praC	EC: 5.3.2.6	4-Oxalocrotonate tautomerase
praH	EC:4.1.1.45	5-Carboxy-2-hydroxymuconate-6-semialdehyde decarboxylase
ligA	EC: 1.13.11.8	Protocatechuate 4,5-dioxygenase, alpha chain
ligB	EC: 1.13.11.8	Protocatechuate 4,5-dioxygenase, beta chain
ligC	EC: 1.1.1.312	4-Carboxy-2-hydroxymuconate-6-semialdehyde dehydrogenase
ligI	EC: 3.1.1.57	2-Pyrone-4,6-dicarboxylic acid hydrolase
ligJ	EC: 4.2.1.83	2-Keto-4-carboxy-3-hexenedioate hydratase
ligK	EC: 4.1.3.17	4-Carboxy-4-hydroxy-2-oxoadipate aldolase
ligR	NA	LysR family transcriptional regulator

NA: Not available.

Table S5. Statistical analysis (multivariate ANOVA) performed on each treatment group.

**Treatment group**	**Interaction**	**Degradation**	**Decolouration**
**Lignin concentrations**	Between two consortia	**	**
	Among five lignin concentrations	-	**
	Between consortia and treatment group	-	**
**Temperatures**	Between two consortia	**	**
	Among three temperatures	*	*
	Between consortia and treatment group	-	-
**Salt concentrations**	Between two consortia	**	**
	Among six salt concentrations	*	**
	Between consortia and treatment group	-	-
**pH values**	Between two consortia	**	**
	Among five pH values	*	**
	Between consortia and treatment group	-	-

Note: ‘-’: $p > 0.05$ (not significant); ‘*’: $p < 0.05$ (significant); ‘**’: $p < 0.001$

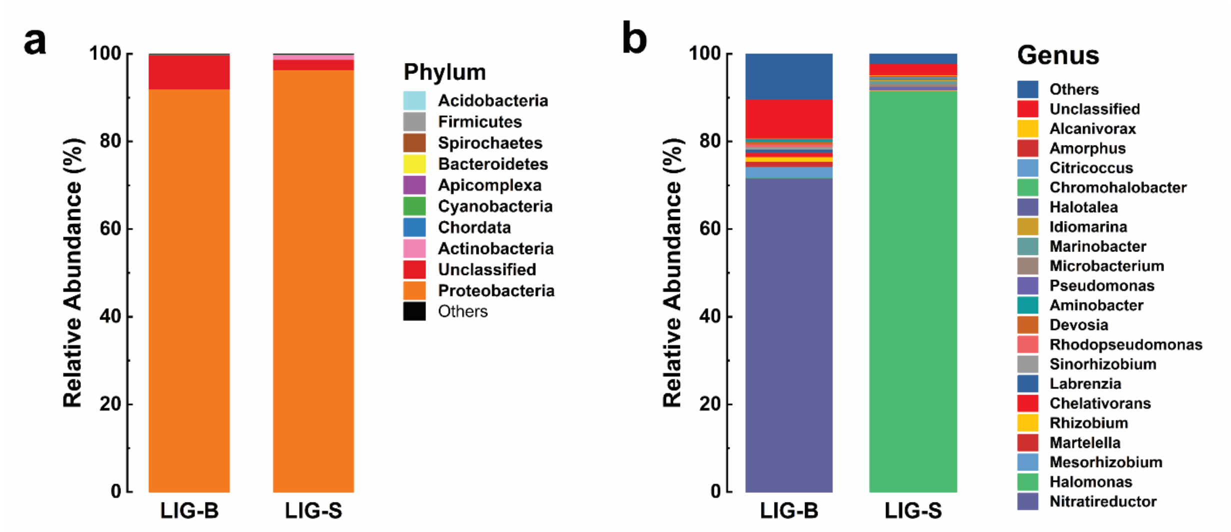

Fig. S1. Bacterial composition and relative abundance of consortia LIG-B and LIG-S at the (a) phylum and (b) genus levels. Phyla in consortium LIG-B: Proteobacteria (91.94%), Actinobacteria (0.04%), Chordata (0.08%), Cyanobacteria (0.8%), Apicomplexa (0.03%), Bacteriodetes (0.02%), Spirochaetes (0.2%), Firmicutes (0.1%), Unclassified (7.78%). Phyla in consortium LIG-S: Proteobacteria (96.36%), Actinobacteria (1.08%), Chordata (0.01%), Cyanobacteria (0.02%), Bacteriodetes (0.02%), Firmicutes (0.09%), Acidobacteria (0.01%), Others (0.06%), Unclassified (2.37%). Genera in consortium LIG-B: *Nitratireductor* (71.59%), *Mesorhizobium* (2.46%), *Martelella* (1.16%), *Rhizoboium* (1.05%), *Chelativorans* (1.01%), *Labrenzia* (0.78%), *Sinorhizobium* (0.59%), *Rhodopseudomonas* (0.58%), *Devosia* (0.51%), *Aminobacter* (0.49%), Halomonas (0.21%), *Pseudomonas* (0.15%), *Amorphus* (0.08%), *Microbacterium* (0.04%), *Marinobacter* (0.04%), *Idiomarina* (0.02%), Unclassified (8.86%) and Others (10.38%). Genera in consortium LIG-S: *Halomonas* (91.55%), *Pseudomonas* (0.78%), *Microbacterium* (0.71%), *Marinobacter* (0.64%), *Idiomarina* (0.34%), *Halotalea* (0.25%), *Chromohalobacter* (0.18%), *Citricoccus* (0.17%), *Amorphus* (0.16%), *Alcanivorax* (0.14%), *Mesorhizobium* (0.01%), *Rhizoboium* (0.11%), *Chelativorans* (0.01%), *Sinorhizobium* (0.01%), *Rhodopseudomonas* (0.01%), Unclassified (2.69%) and Others (2.24%).

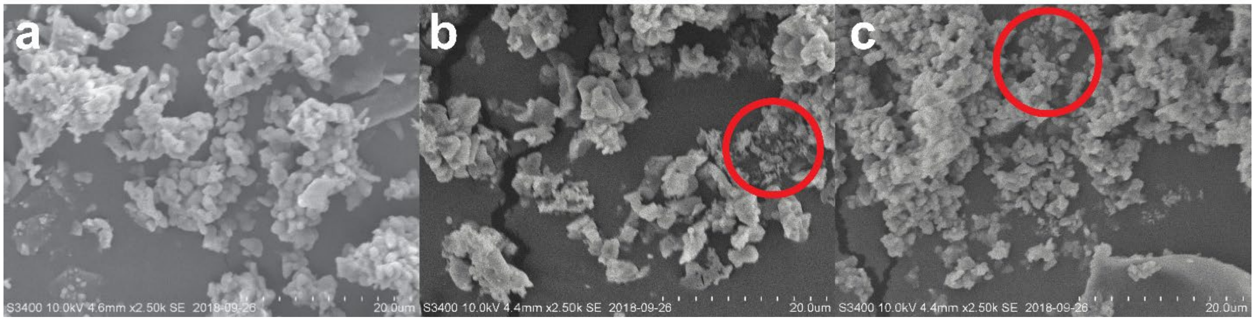

Fig. S2. **Scanning electron microscopy (SEM) images of alkali lignin.** (a): alkali lignin without inoculation (the negative control); (b): alkali lignin treated by consortium LIG-B; (c): alkali lignin treated by consortium LIG-S. Samples were treated for 7 days. The red circles indicate the more irregular and smaller fragments, resulted from alkali lignin depolymerization by each consortium, compared with the densely and aggregated fragments in the negative control.

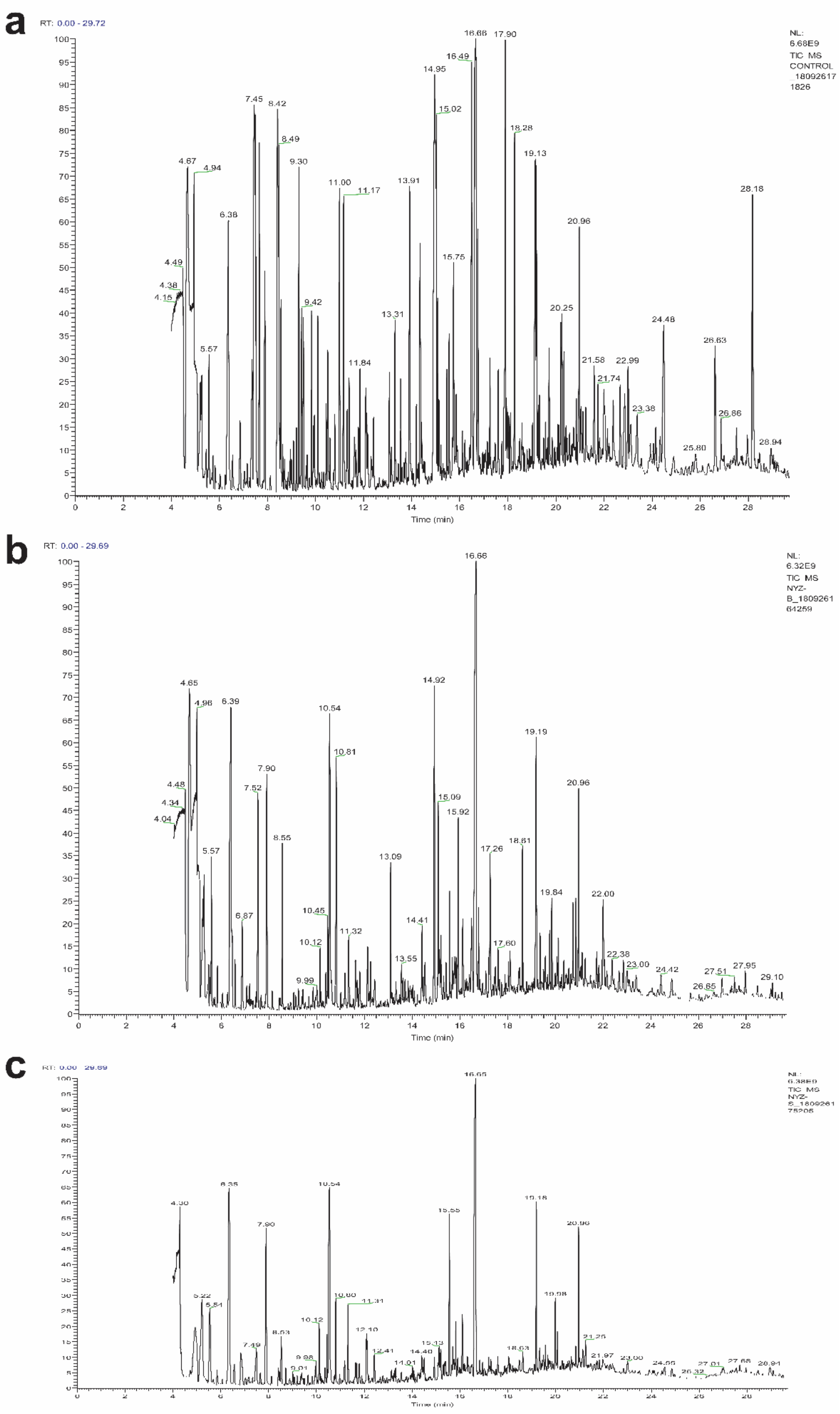
a
RT: 0.00 - 29.72
NL:
6.68E9
TIC MS
CONTROL
_18092617
1826
4.15 4.38 4.49 4.67 4.94 5.57 6.38 7.45 8.42 8.49 9.30 9.42 11.00 11.17 11.84 13.31 13.91 14.95 15.02 15.75 16.49 16.66 17.90 18.28 19.13 20.25 20.96 21.58 21.74 22.99 23.38 24.48 25.80 26.63 26.86 28.18 28.94
Time (min)
b
RT: 0.00 - 29.69
NL:
6.32E9
TIC MS
NYZ-
B_1809261
64259
4.04 4.34 4.48 4.65 4.96 5.57 6.39 6.87 7.52 7.90 8.55 9.99 10.12 10.45 10.54 10.81 11.32 13.09 13.55 14.41 14.92 15.09 15.92 16.66 17.26 17.60 18.61 19.19 19.84 20.96 22.00 22.38 23.00 24.42 26.85 27.51 27.95 29.10
Time (min)
c
Time (min)

Fig. S3. Total ion chromatograph of TMS derivatives of compounds by gas chromatography. (a) alkali lignin (negative control), (b) alkali lignin treated by consortium LIG-B, (c) alkali lignin treated by consortium LIG-S.

References

1. Song D, Zhang Y, Liu J, Zhong H, Zheng Y, Zhou S, Yu M, Todd JD, Zhang XH. 2020. Metagenomic insights into the cycling of dimethylsulfoniopropionate and related molecules in the eastern china marginal seas. Front Microbiol 11:157.
2. Rasigraf O, van Helmond NAGM, Frank J, Lenstra WK, Egger M, Slomp CP, Jetten MSM. 2020. Microbial community composition and functional potential in Bothnian Sea sediments is linked to Fe and S dynamics and the quality of organic matter. Limnol Oceanogr 65:S113–S133.
3. Nathani NM, Dave KJ, Vatsa PP, Mahajan MS, Sharma P, Mootapally C. 2021. 309 metagenome assembled microbial genomes from deep sediment samples in the Gulfs of Kathiawar Peninsula. Sci Data 8:1-8.
4. Mizuno CM, Rodriguez-Valera F, Ghai R. 2015. Genomes of planktonic acidimicrobiales: Widening horizons for marine Actinobacteria by metagenomics. mBio 6:02083-14.
5. Bech PK, Lysdal KL, Gram L, Bentzon-Tilia M, Strube ML. 2020. Marine Sediments Hold an Untapped Potential for Novel Taxonomic and Bioactive Bacterial Diversity. mSystems 5:e00782-20.
6. Cabello-Yeves PJ, Callieri C, Picazo A, Mehrshad M, Haro-Moreno JM, Roda-Garcia JJ, Dzhembekova N, Slabakova V, Slabakova N, Moncheva S, Rodriguez-Valera F. 2021. The microbiome of the Black Sea water column analyzed by shotgun and genome centric metagenomics. Environ Microbiome 16:5.
